# Dynamic postcricoid venous malformation causing episodic airway obstruction: a multidisciplinary report

**DOI:** 10.1007/s11282-026-00940-8

**Published:** 2026-06-11

**Authors:** Joshua Rapp, Brian Kulbersh, Junaid Raja

**Affiliations:** 1 Department of Interventional Radiology Heersink School of Medicine, University of Alabama at Birmingham, 1720 2nd Avenue South, Birmingham, AL 35294 USA; 2 Department of Otolaryngology Heersink School of Medicine , University of Alabama at Birmingham , 1720 2nd Avenue South, Birmingham, AL 35294 USA; 3 Children’s of Alabama , 1720 2nd Avenue South, Birmingham, AL 35294 USA

**Keywords:** Pediatric airway obstruction, Postcricoid, Dynamic airway lesion, Sclerotherapy, Venous malformation

## Abstract

**Purpose:**

To describe a rare case and presentation of a dynamic postcricoid hypopharyngeal venous malformation in a young child, including its diagnostic work-up, and successful multidisciplinary treatment with percutaneous ethanol sclerotherapy.

**Materials and Methods:**

Under general anesthesia, the patient was intubated, and the airway was evaluated by otolaryngology using a Miller laryngoscope with Hopkins rod visualization of the postcricoid region. A controlled Valsalva maneuver was performed by anesthesia to distend the venous malformation and enhance visualization. Interventional radiology then directly punctured the lesion with a 21-gauge needle, injected contrast to confirm intralesional placement, and performed sclerotherapy with 1.5 cc of 98% ethanol.

**Results:**

Sclerotherapy was technically and clinically successful. Follow-up laryngoscopy demonstrated complete resolution of the patient’s symptoms along with resolution of the venous malformation without complications.

**Conclusion:**

This case highlights the importance of multidisciplinary collaboration in managing dynamic airway vascular anomalies and demonstrates the safety and efficacy of percutaneous alcohol sclerotherapy for pediatric airway venous malformations.

## Introduction

Venous malformations are congenital lesions characterized by abnormal venous channels that enlarge with increased venous pressure and do not involute spontaneously [1]. In particular, venous malformations of the pediatric airway are rare and potentially life-threatening lesions that require precise diagnosis and patient-tailored treatment plans depending on the lesion’s dynamic physiological properties. For these specific vascular anomalies, the ultimate priority is evaluation of the risk for the airway and anticipating complications with management [2, 4]. Conventional imaging, such as US/CT, are situationally helpful in evaluating venous malformations, but can fail to capture dynamic lesions in a resting low flow or static state [3]. Therefore, conventional imaging can compromise accurate diagnosis of dynamic vascular lesions and direct exploratory visualization often provides the best understanding for treatment options [4].

These airway vascular malformations can be classified based on severity of symptoms to determine the state of medical emergency. Mild-moderate states can present with increased work of breathing while more severe cases can lead to cyanosis, with stridor or stertor giving clues to anatomical airway obstruction [2]. We present a rare case of a categorically severe postcricoid venous malformation in a two-year-old child that caused episodic airway obstruction only during agitation. This case highlights the shortcomings of conventional imaging in dynamic vascular anomalies, the need for direct visualization and procedural adaptability for proper lesion diagnosis, and the importance of interdisciplinary collaboration in the management of dynamic pediatric airway vascular anomalies.

### Case report

#### Patient presentation

**Fig. 1:**
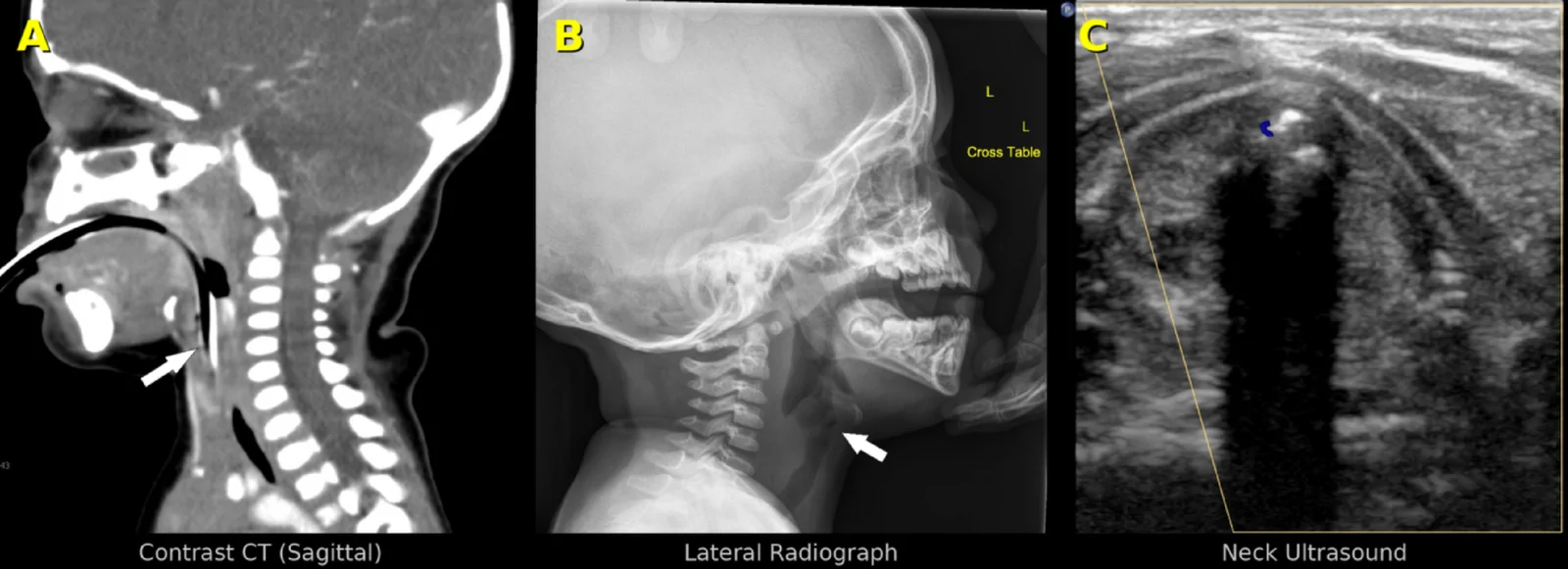
Pre-procedural imaging performed under resting/anesthetized conditions. **A** Contrast-enhanced CT of the neck, sagittal reconstruction. Arrow denotes the postcricoid region, where no discrete soft tissue mass or vascular abnormality is identified at rest. **B** Lateral plain radiograph of the cervical soft tissues. Arrow indicates the postcricoid soft tissue, demonstrating nonspecific, unremarkable appearance without evidence of mass effect. **C** Cervical ultrasound with color Doppler. The postcricoid region shows no discernible vascular engorgement or abnormal flow signal demonstrated

**Fig. 2:**
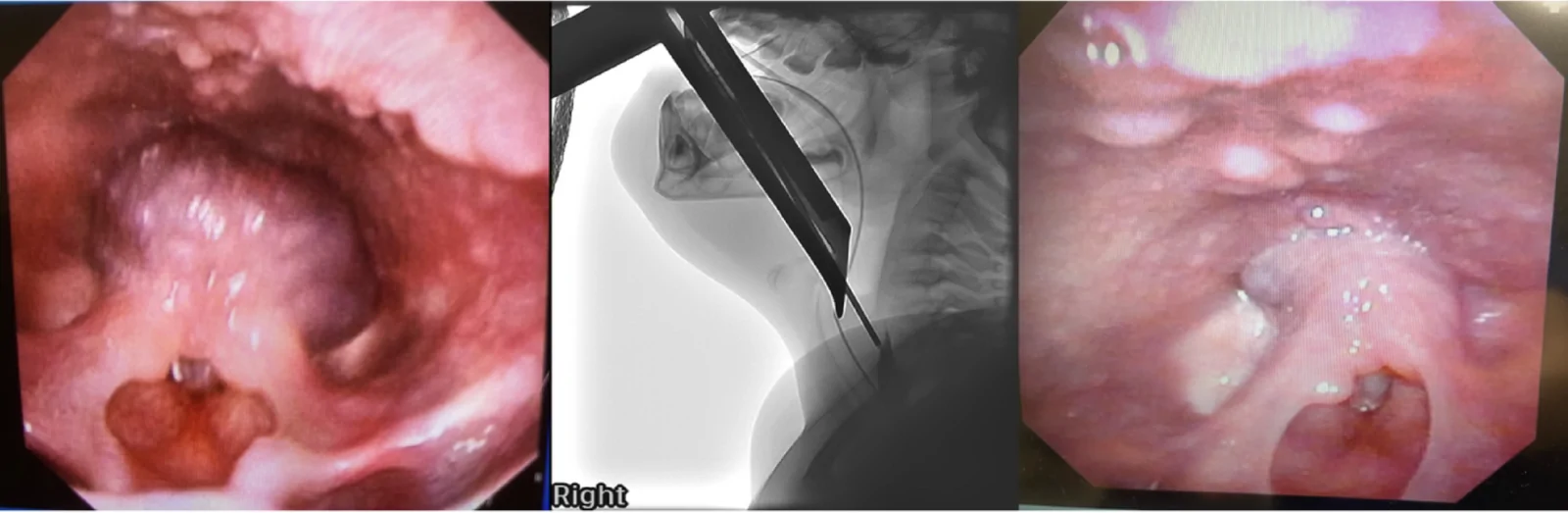
**A** Pre-interventional laryngoscopy demonstrating an engorged postcricoid venous malformation. **B** Intraoperative fluoroscopic image showing direct percutaneous puncture and ethanol sclerotherapy. **C** Two-month postoperative follow-up laryngoscopy demonstrating resolution of the venous malformation

Written informed consent for publication of this case and associated images was obtained from the patient’s legal guardian. A two-year-old male presented with transient episodes of respiratory distress characterized by cyanosis during states of agitation. Pre-procedural imaging was obtained. Across all three modalities, imaging was performed under resting conditions with the patient hemodynamically relaxed, and the lesion remained entirely occult (Fig. 1a–c) [3]. Because conventional imaging did not aid in the diagnostic process, the vascular anomaly was investigated via flexible laryngoscopy and direct laryngoscopy with bronchoscopy, providing a safe further diagnostic workup and full visualization of the lesion (Fig. 2a) [4]. The dynamic lesion was diagnosed as a venous malformation and was shown to engorge predictably with elevated venous pressure, explaining why obstructive symptoms arose exclusively during agitation but appeared benign on standard imaging. While direct visualization with laryngoscopy aided in diagnosis and treatment decisions, it did not provide an optimal physiological environment for treatment itself. In general, airway venous malformation manipulation and removal presented with risks given the location, as traditional surgical excision was associated with an unacceptably high possibility of complex reconstruction, incomplete lesion removal, and airway stricturing [5]. After careful consideration, a multidisciplinary treatment strategy was selected, utilizing percutaneous sclerotherapy under the combined expertise of otolaryngology, interventional radiology, and anesthesia.

### Multidisciplinary procedural design

The patient was placed in supine position and under general anesthesia to minimize inadvertent patient movements. The patient was then intubated via a 4 mm endotracheal tube with 0.5mL of air in the cuff. Oxymetazoline 0.05% was applied to cottonoids and placed in the patient’s nasal cavity to induce mucosal vasoconstriction while topical anesthetic was administered to the laryngotracheal mucosa prior to instrumentation. The airway was carefully examined by otolaryngology using a Miller laryngoscope in combination with a Hopkins rod telescope, allowing high-resolution, magnified visualization of the postcricoid region and surrounding airway structures. To reproduce physiologic engorgement of the vascular malformation, anesthesia performed a controlled Valsalva maneuver, resulting in distention of the lesion and improved visualization. This allowed for interventional radiology to advance a 21G needle into the nidus of the venous malformation. Once intravascular needle placement was confirmed by blood return, a short connector primed with iodinated contrast was injected to delineate the vascular anatomy (Fig. 2b). The selected sclerosant was 98% ethanol given lesion flow and characteristics, and documented success of ethanol with venous malformations [6]. Interventional radiology then slowly injected 1.5 cc of ethanol with the distribution of sclerosant within the malformation visualized fluoroscopically through contrast displacement. After the needle was removed, there was no noted oozing of blood from the puncture site nor any blanching of the mucosa or ulceration. Additionally, venous malformation thrombosis was confirmed by lack of distension of the sclerotherapy area with repeated Valsalva maneuver. Following completion of the procedure, the patient was cleaned and general anesthesia was reversed prior to transfer to the recovery area.

### Postoperative report

The patient was admitted to the PICU postoperatively for respiratory monitoring and pain dose management where he had a transient episode of stridor without desaturation. Originally, steroids were avoided to preserve natural inflammatory response but were later deemed necessary due to the patient’s stridor [7]. On day one postoperatively, the patient’s airway inflammation was managed with a multimodal corticosteroid regimen, including injected and nebulized dexamethasone, and he was able to be downgraded to the floor without any subsequent postoperative complications. Eventually, the patient was discharged within forty-eight hours on a five-day course of oral suspension amoxicillin/clavulanate (600 mg-42.9/5mL), twice a day, and a continued three-day course of oral suspension prednisolone (15 mg/5mL), 2 mL in the morning. The patient denied any recurrence of symptoms and clinical follow-up confirmed resolution of the venous malformation via laryngoscope with Valsalva maneuver. Visualization of the sclerotherapy site and surrounding airway structures demonstrated healthy postoperative mucosal healing without evidence of ulceration, stricturing, necrosis, or other treatment-related adverse effects (Fig. 2c).

## Discussion

Pediatric airway venous malformation represents an uncommon vascular anomaly and requires care teams to be vigilant in precise diagnosis and treatment planning given these cases can be technically challenging. Percutaneous sclerotherapy is established as an effective minimally invasive treatment modality for venous malformations [6]. However, this case report comments on the safety of percutaneous sclerotherapy specifically in pediatric airway venous malformations. When care teams are approaching vascular anomalies, considerations should be tailored towards lesion characteristics and anatomy. The first step should be visualizing these lesions in their fullest extent before deciding between surgical excision or sclerotherapy [4, 5].

This study demonstrates that conventional static imaging may underestimate the true clinical significance of venous malformations [3]. Furthermore, comprehensive visualization and treatment of these vascular anomalies under dynamic stressors requires multidisciplinary collaboration [5]. The focus of collaboration should be optimizing lesion delineation and access. Intraoperative manipulation of vascular anomalies requires physiologic strategies tailored to lesion type and anatomic location. While controlled Valsalva was effective for this low-flow venous malformation, other anomalies may require different anesthetic techniques based on flow characteristics. Beyond physiological considerations, the lesion must be exposed in a procedurally relevant fashion to provide safe and effective treatment [4, 5]. This case showcased a multidisciplinary approach relying on the anatomical expertise of otolaryngology to visualize the airway, and the procedural expertise of interventional radiology with sclerotherapy.

Finally, patients must be carefully monitored postoperatively given the potential for swelling and airway obstruction, especially with venous malformations [2]. Steroids should be avoided if possible to preserve the natural healing and inflammatory processes of ethanol sclerotherapy but complications including stridor, such as in this case, may warrant usage [7]. Postoperative management should be determined on a case-by-case basis, especially when dealing with lesions near airway structures.

## Conclusion

Controlled provocation combined with direct laryngoscopy visualization and percutaneous ethanol sclerotherapy allowed safe and effective management of a venous malformation that was otherwise occult under standard conditions. In this case, a rare pediatric postcricoid venous malformation was misrepresented with conventional imaging and required further workup. This approach does not propose a systematic algorithm but rather highlights key multidisciplinary considerations for accurately diagnosing and managing dynamic vascular airway anomalies.

## Data Availability

No datasets were generated or analysed during the current study.

## References

[1] International Society for the Study of Vascular Anomalies (ISSVA). *ISSVA classification for vascular anomalies*. Approved at the 20th ISSVA Workshop, Melbourne, April 2014; last revision May 2018. Available at: https://www.issva.org/classification

[2] Gorostidi F, Glasson N, Salati V, Sandu K. Pediatric vascular anomalies with airway compromise. J Oral Pathol Med. 2022;51(10):888–96. 10.1111/jop.13297. PMC10084185 35347777

[3] Behravesh S, Yakes W, Gupta N, et al. Venous malformations: clinical diagnosis and treatment. Cardiovasc Diagn Ther. 2016;6(6):557–69. 10.21037/cdt.2016.11.10. PMC5220204 28123976

[4] Clymer MA, Fortune DS, Weiner RL, Auger SR, Perkins JA, Inglis AJ Jr. Venous malformation of the pediatric airway. Int J Pediatr Otorhinolaryngol. 1997;39(1):69–79. 10.1016/s0165-5876(96)01456-6.

[5] Gallant SC, Chewning RH, Orbach DB, Trenor CC, Cunningham MJ. Contemporary management of vascular anomalies of the head and neck—part 1: vascular malformations: a review. JAMA Otolaryngol Head Neck Surg. 2021;147(2):197–206. 10.1001/jamaoto.2020.4353. 33237296

[6] Burrows PE, Mason KP. Percutaneous treatment of low flow vascular malformations. J Vasc Interv Radiol. 2004;15(5):431–45. 10.1097/01.rvi.0000124949.24134.cf. 15126652

[7] Cao J, Liu J, Zhang X, Wang Z. A systematic review and network meta-analysis of the effectiveness of sclerotherapy for venous malformation. J Vasc Surg Venous Lymphat Disord. 2023;11(1):210–e2183. Epub 2022 Sep 28. PMID: 36179784. 10.1016/j.jvsv.2022.08.004 36179784

